# Abnormal Elevated CA 19-9 in the Dermoid Cyst: A Sign of the Ovarian Torsion?

**DOI:** 10.1155/2013/860505

**Published:** 2013-06-11

**Authors:** Burcu Artunc Ulkumen, Asli Goker, Halil Gursoy Pala, Sercin Ordu

**Affiliations:** Department of Obstetrics and Gynecology, Celal Bayar University, 45010 Manisa, Turkey

## Abstract

Dermoid cyst is the most common germ cell tumor of the ovary containing various tissue elements. Ovarian torsion is a common complication of which ultrasonographic diagnosis is confusing. We report here a 14-year-old adolescent with painless torsion of the ovary including dermoid cyst and with abnormal elevated CA 19-9 serum levels. Elevated CA 19-9 level may be related to ovarian torsion and may predict the extent of tissue necrosis.

## 1. Introduction

Dermoid cyst, also called as mature cystic teratoma (MCT) or benign cystic teratoma of the ovary, is the most common germ cell tumor of the ovary in reproductive aged women [1]. It accounts for 10–25% of all ovarian neoplasms and 60% of all benign ovarian tumors [2]. Malignant transformation occurs in only 1–3% of cases, especially in postmenopausal women. 15% of cases develop torsion; however, rupture is rare probably due to thick cyst wall. It is mostly common in women aged between 20 and 40 years [3]. Ultrasonography is the main diagnostic tool. However, the diagnosis may be confusing as the cyst contains various tissue elements.

Carbohydrate antigen 19-9 (also called as cancer antigen 19-9; sialylated Lewis (a) antigen) is mainly increased in gastrointestinal system tumors. However, it can be detected in other malignancies and in some benign conditions as well. To the best of our knowledge, the present study is reporting the highest serum level of CA 19-9 in association with dermoid cyst.

## 2. Case

A 14-year-old virgin adolescent was admitted to our clinic because of pelvic pain lasting for 1 week. Abdominopelvic ultrasonography revealed a lobulated cystic lesion with a diameter of 11 cm in the right adnexa. Abdominopelvic MR revealed a 16 cm cystic lesion consisting of heterogeneous solid structures. The left ovary and other intra-abdominal structures were normal. Tumor markers were as follows: CEA: 1.90 U/mL, AFP: 0.94 U/mL, CA 15-3: 13.4 U/mL, CA 19.9: 1983 U/mL, and CA 125: 217 U/mL.

**Figure 1:**
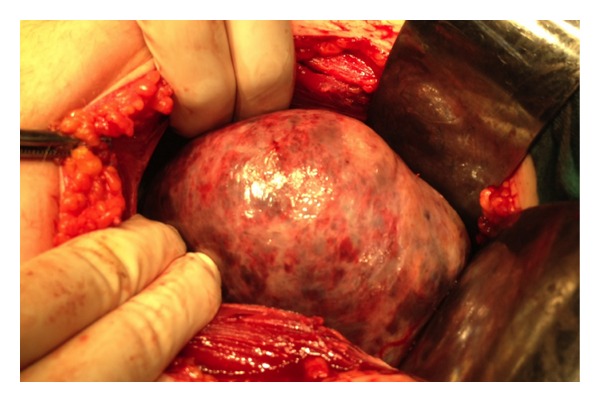
Intra-operative view of the ovarian torsion with necrosis containing dermoid cyst.

Another possible gastrointestinal system pathology was ruled out by imaging modalities. Pfannenstiel incision was made, and right ovarian torsion with necrosis was detected (Figure 1). Right salpingooophorectomy was performed, and frozen section revealed dermoid cyst. Pathological evaluation was compatible with dermoid cyst and the ovarian torsion.

Postoperatively tumor markers return to normal levels.

## 3. Discussion

Dermoid cysts are 10–25% of all ovarian tumors (1 = 14,2). 60% of them are 5–10 cm in diameter [1]. Bilaterality is found in 10% of cases. If unilateral, it is found more commonly in the right adnexa, as in our case [1]. They contain mature elements of 3 germ cell layers. Typical findings of ultrasonographic examination include echogenic lines in the cyst fluid and shadowing. However, it is very difficult to notice the sonographic findings of torsion in an ovary comprising dermoid cyst. Due to shadowing pattern of these tumors, ultrasonography may not be useful to recognize the torsion. Furthermore, normal color Doppler findings may not exclude the diagnosis of the torsion. Supplemental means may be helpful in differential diagnosis of torsion due to the need for early recognition and treatment. Few tumor markers such as CA 19-9 and CA 125 have been the topics of recent studies pointing that CA 19-9 may be related to the existence of the dermoid cyst [3–6].

Approximately 1–3% of dermoid cysts can be complicated with malignant transformation. However, CA 125 and CA 19-9 levels appear inconsequential with squamous cell carcinoma arising from dermoid cyst [7].

In a study consisting of 215 cases, CA 19-9 levels were elevated in 40% of selected population with mature cystic teratoma. Mean serum levels of CA 19-9 were 83.5 + 179.2 U/mL [3]. CA 125 levels with the mean 26.2 + 29.9 U/mL were significantly higher in patients with elevated CA 19-9 [3]. In an another study including 250 patients, 31 cases had CA 19-9 above 101 U/mL [6]. Serum levels of CA 19-9 were correlated with the tumor size [3–6]. Bilaterality was debated regarding the extent of elevation [3, 4]. Correlation between bilaterality and elevation of CA 19-9 was found only in one study [4]. Concomitant elevation of CA 125 and CA 19-9 was only in 3% (5/163) cases [5]. However, the clinical significance with elevated CA 19-9 levels is still controversial. A recent study by Kyung et al. postulated that CA 19-9 may be a predictor of the ovarian torsion. The mean serum CA 19-9 levels of 21 cases with ovarian torsion were 37.2 U/mL (range: 14.1–570.0), whereas mean serum CA 19-9 levels of 21 cases without ovarian torsion were 20.2 U/mL (*P* = 0.0062) [6]. Regarding these results, our patient had much higher level of CA 19-9 with 1983 U/mL, probably the highest serum level related to dermoid cyst reported up to date. We suggest that abnormal elevated CA 19-9 may be related to the torsion of the ovary. Furthermore, it may be related to the extent of necrosis of the ovary. Detorsion was not possible in the present case due to the diffuse necrosis of the ovary. The extent of necrosis and the modality of the surgery (detorsion, oophorectomy) were not mentioned in the study by Kyung et al. [5].

Elevated CA 19-9 levels may also be a result of the rupture of the dermoid cyst leading to leakage from cyst wall into the bloodstream [2]. A weakened cyst wall due to large diameter of the cyst may be another cause of the elevated CA 19-9 level [2].

The elevated CA 125 level in dermoid cyst may be due to peritoneal involvement such as pelvic inflammatory disease or endometriosis [1]. In our case, with the torsion of the ovary, an inflammatory process may be the cause of great elevation of CA-125. The cyst wall may become weaker and weaker by the torsion leading to greater increase in serum levels of Ca 19-9.

In conclusion, high levels of CA 19-9 and CA-125 and the rapid increase in the diameter of the cyst are not always associated with malignancy. However, a detailed preoperative evaluation is needed. Due to the need of early detection of ovarian torsion, CA 19-9 may be a good marker particularly for ovarian torsion and the extensity of ovarian necrosis. However, larger studies are needed to confirm this hypothesis.
